# Case report and literature review: Giant retroperitoneal cystic lymphangioma

**DOI:** 10.3389/fsurg.2023.1074067

**Published:** 2023-01-17

**Authors:** Tieshan Su, Chaoyuan Li, Bin Song, Defeng Song, Ye Feng

**Affiliations:** ^1^ Departments of Gastrointestinal Colorectal Anus Surgery, China-Japan Union Hospital, Jilin University, Changchun, China; ^2^ Departments of Orthopedics Surgery, China-Japan Union Hospital, Jilin University, Changchun, China

**Keywords:** cystic lymphangioma, retroperitoneal tumor, surgical resection, case report, literature review

## Abstract

**Background:**

Cystic lymphangioma is a rare benign tumor of the lymphatic system, which is most commonly observed in the neck, head and armpit.Less than 5% of lymphangiomas occur in the abdominal cavity and even less in the retroperitoneum.

**Case description:**

A 65-year-old male patient was diagnosed with an “abdominal mass that had persisted for 1 year, accompanied by abdominal pain, abdominal distension and dyspnea for 7 days”. After abdominal computerd tomography, a giant multilobed abdominal lymphangioma was suspected, which squeezed the intestinal canal and was closely related to the inferior vena cava. The patient underwent an exploratory laparotomy, during which, it was found that the tumor formed extensive adhesions to the transverse colon, small intestine and pelvic wall, and enveloped the abdominal aorta, superior mesenteric artery, inferior mesenteric artery and inferior vena cava to varying degrees. It was diffcult to remove the cyst completely. Postoperative pathology confirmed the diagnosis of retroperitoneal cystic lymphangioma. The patient recovered well after the operation, was eating normally by 5 days postoperatively,and was discharged 10 days postoperatively.The patient was followed up 1 month after postoperatively and no evidence of recurrence was observed.

**Conclusion:**

In this case, we report a patient with giant retroperitoneal cystic lymphangioma who underwent exploratory laparotomy combined with preoperative abdominal computerd tomography and acute abdominal pain, abdominal distension and dyspnea. Because of the large volume of the tumor and its close relationship with the superior mesenteric artery and other blood vessels, the surgeon used scissors to separate the tumor sharply and removed the whole tumor completely.

## Introduction

Cystic lymphangioma is a non-cancerous vascular malformed conjunctival tumor consisting of thin-walled cysts. It is a rare benign tumor, which is most commonly observed in the neck, head,and armpit, while >5% of lymphangiomas occur in the abdominal cavity, including the mesentery and the greater omentum.The occurrence in the retroperitoneum is even rarer. Although cystic lymphangioma can occur at any age, it is mainly observed in children, in whom the prognosis is good (1, 2). In adults, cystic lymphangiomas account for approximately 7% of abdominal cysts (3). Retroperitoneal cystic lymphangioma is usually asymptomatic, but in some cases it is characterized by acute abdominal pain and distension caused by nausea, vomiting or intestinal obstruction. The tumor can be identified by imaging examinations such as ultrasound and computed tomography (CT), however histopathological examination remains the gold standard. Complete surgical resection of the tumor is the first choice for treatment (4–6). Here, we report the clinical features, diagnosis, treatment and prognosis of a giant retroperitoneal cystic lymphangioma in a 63-year-old male patient. And this study is in accordance with the principles of CARE guidelines (7).

## Case description

**Figure 1:**
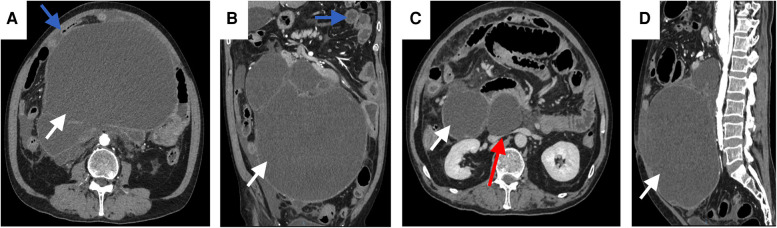
Abdominal enhanced CT axial arterial phase (**A**), coronary arterial phase (**B**), axial venous phase (**C**),and sagittal arterial phase (**D**) CT images show a retroperitoneal cystic mass (white solid arrow), extending along the mesentery to 24 cm. The tumor oppresses the surrounding intestine (blue solid arrow) and the inferior vena cava (**B**) (red solid arrow).

**Figure 2:**
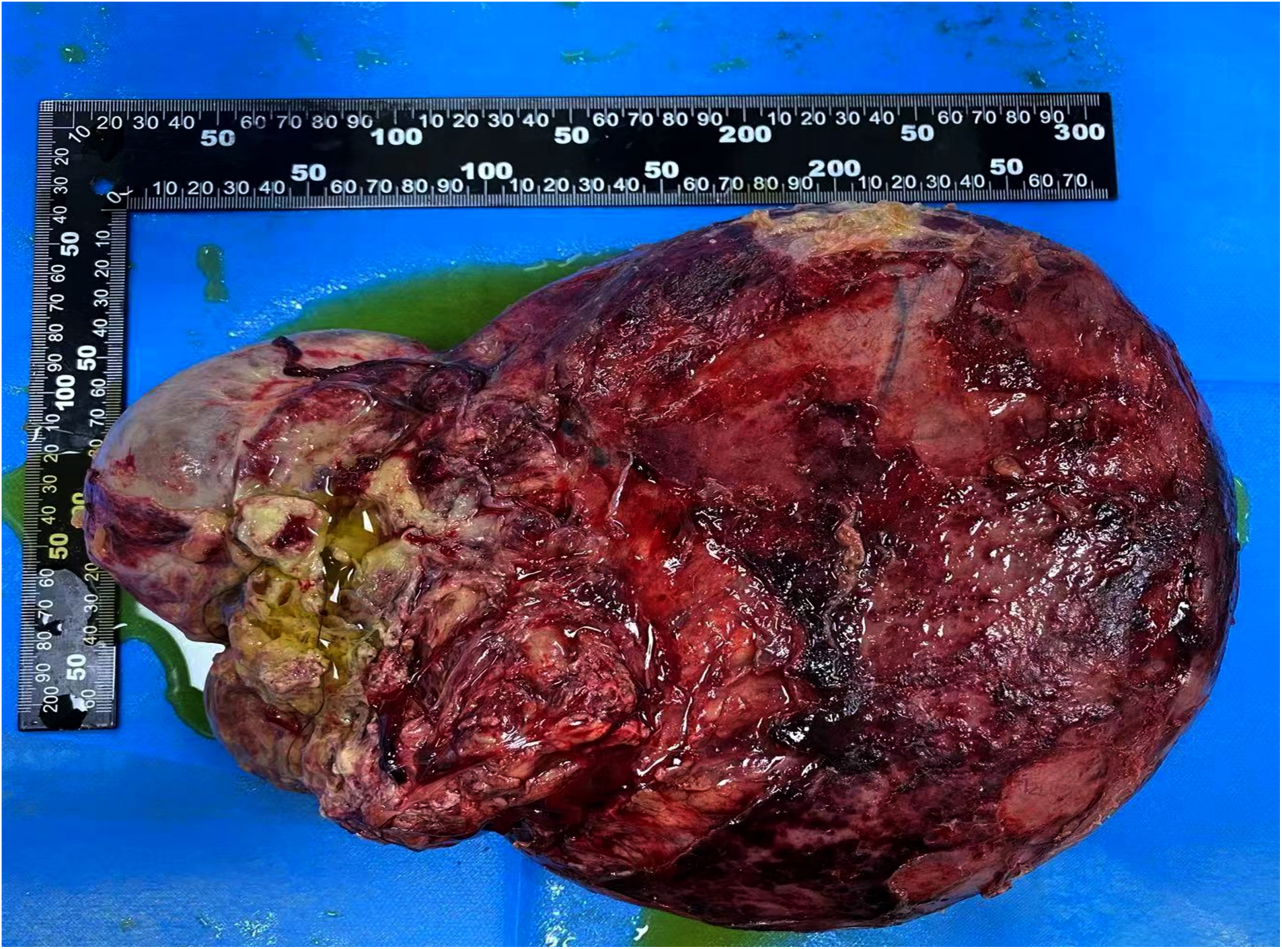
Image of the gross specimen.

**Figure 3:**
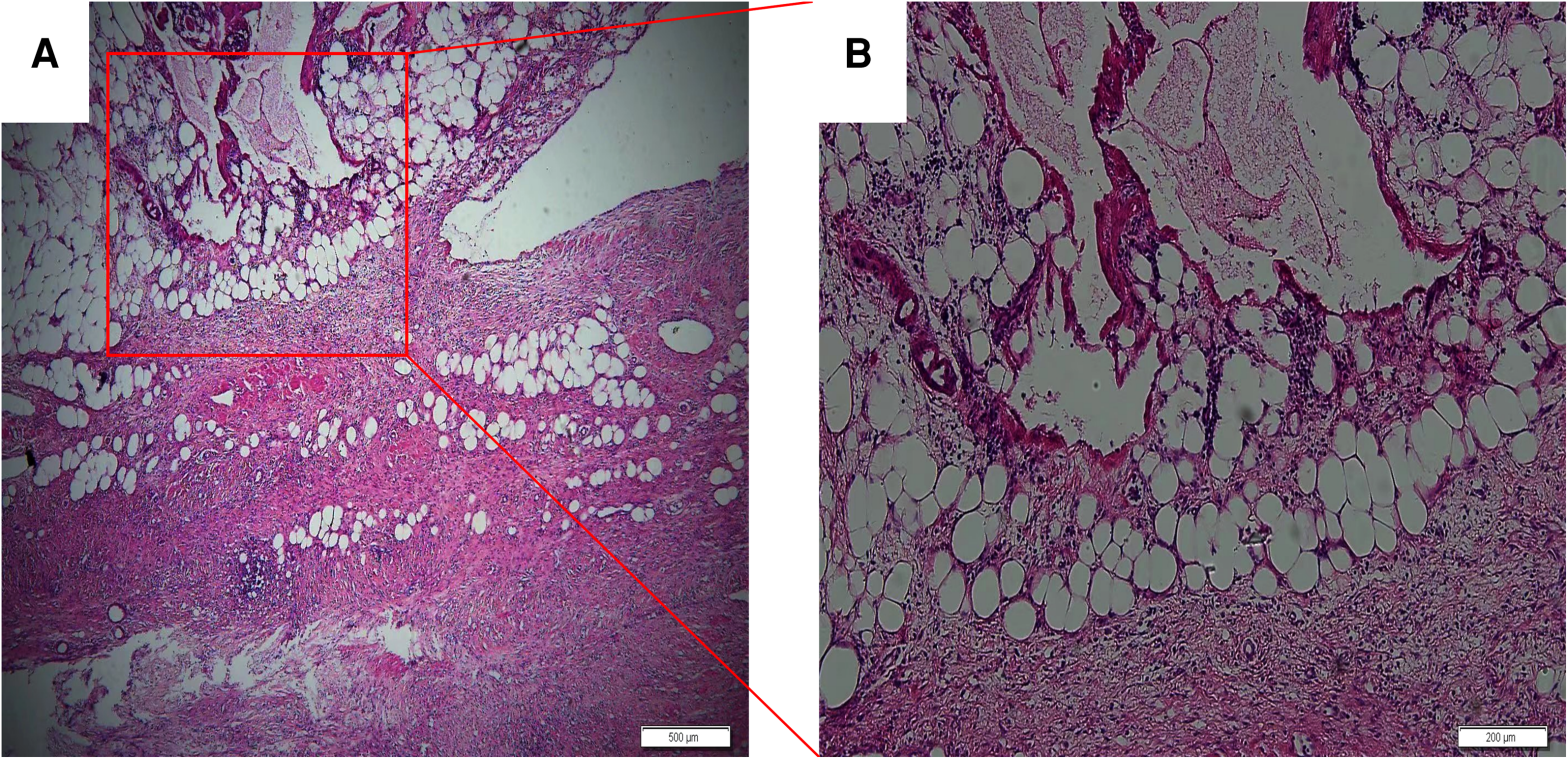
Necrosis and protein exudation in the cystic cavity, obvious proliferation of interstitial vessels and fibrous tissue, cystic lymphatic cavities of different sizes with inflammatory lymphocyte infiltration and lymphoid nodules; (**A**) hematoxylin and eosin (HE) × 40, scale 500 μm; (**B**) HE × 100, scale 200 μm.

The patient, a 65-year-old man, was diagnosed with abdominal swelling that had persisted for 1 year,and was accompanied by abdominal pain, abdominal distension and dyspnea for 7 days. A year prior, the patient found an"abdominal cystic mass (approximately 25 cm × 1.6 cm × 22 cm)” and attended an other hospital. He was admitted to the local hospital 7days prior due to sudden intermittent diffuse abdominal pain with abdominal distension, dyspnea, no fever, nausea, and vomiting. Abdominal CT suggested an “abdominal mass and intestinal obstruction”, and the symptoms did not improve. The patient was admitted to the hospital for further diagnosis and treatment. The patient had had hypertension for 10 years and regularly took levamlodipine besylate tablets for 5 mg/days,which controlled his blood pressure within the normal range. A cardiac stent operation was performed 4 months prior because of myocardial infarction. Physical examination showed: no obvious heart abnormalities,although shortness of breath, obvious abdominal swelling, frog belly, no gastrointestinal type and peristaltic wave were noted,as well as a large mass that could be felt asoccupying most of the abdominal cavity. Regarding tumor markers: CA125:65.47 U/ml (reference value < 35 U/ml), other tumor markers were not abnormal. Abdominal enhanced CT: huge cystic space in the pelvis and abdominal cavity, with an irregular shape, linear septum, (approximately 24.2 cm × 16.7 cm × 27.8 cm), extending along the mesentery of the small intestine (Figures 1A–D). In contrast-enhanced scan, the cystic components were not enhanced, the septum was uniformly enhanced, the intestinal canal was displaced laterally under pressure (Figures 1A, Magi B), and the inferior vena cava was narrowed (Figure 1C). The preliminary diagnosis of the nature of abdominal mass pending diagnosis: cystic lymphangioma? An exploratory laparotomy was performed,during which a large cystic mass of approxmately 30 × 28 × 14 cm was observed retroperitoneally. The surface of the mass was smooth, white and red, with a light yellow exudate on the surface. The tumor formed extensive adhesion with the transverse colon, small intestine and pelvic wall, and enveloped the abdominal aorta, superior mesenteric artery, inferior mesenteric artery and inferior vena cava to varying degrees. We decided to surgically remove the cystic mass completely (Figure 2). Postoperative pathology: microscopically, necrosis and protein exudation in the cystic cavity, chronic inflammation with inflammatory granulation, obvious proliferation of interstitial vessels and fibrous tissue, scattered in clusters of smooth muscle bundles and more inflammatory cells, and occasional giant cells, as well as proliferation of small lymphatic vessels outside the cyst wall, consistent with cystic lymphangioma with chronic inflammation (Figures 3A,B).

The patient recovered well and could eat normally 5 days postoperatively. The patient was discharged 10 days after the surgery,at which point,he was in a good condition, with no pain or complications. In the initial recovery stage, he was advised to ingest high-quality protein that was easily absorbed and high in nutrition,and to avoid high-fat, high-fiber, indigestible and absorbed food, The patient was subjected to, regular re-examination and follow-up.

## Discussion

Cystic lymphangioma usually occurs in the head, neck and armpit, mostly in children, with no sex-related difference in incidence. Intraperitoneal lymphangioma is a rare intra-abdominal tumor. Less than 5% of lymphangiomas occur in the abdominal cavity, and the incidence of retroperitoneal tumors is even lower (1, 2). The etiology of cystic lymphangioma is unclear, and infection, lymphatic obstruction and surgery may be associated factors (8). According to the histological type, cystic lymphangioma can be divided into cystic, capillary and cavernous.Likewise, based on its clinical manifestations, it can be divided into macrocytic, microencapsulated, and mixed cysts. Microcapsules can be further subdivided into developing-cell microcapsules and closed-cell microcapsules based on whether the cells are open or not (9).Here, we report a case of retroperitoneal giant cyst lymphangioma, which is consistent with other reports suggesting that the most common retroperitoneal lymphangioma is cystic type (10).

The clinical manifestations of retroperitoneal cystic lymphangioma are non specific and are related to the size and location of the tumor and the location of the surrounding tissues and organs, which makes diagnosis challenging. Initially, the tumor is small but asymptomatic; with the growth of the tumor, nausea, vomiting, abdominal pain and abdominal distension may occur due to compression of the abdominal organs, and in severe cases, acute abdominal pain and intestinal obstruction may even cause rupture, infection, bleeding and torsion of related organs and tissues (11–13). In the current case, abdominal contrast-enhanced CT showed a huge retroperitoneal cystic mass, which occupied most of the abdominal cavity and pelvic cavity and squeezed the small intestine, resulting in abdominal pain, abdominal distension, dyspnea and other symptoms, and was admitted as “abdominal mass, intestinal obstruction”. Cystic lymphangioma should be differentiated from abdominal lymphoma, mesenteric cyst, secondary metastasis of a malignant tumor, tuberculosis, echinococcosis, small intestinal adenocarcinoma and mesenteric related tumors (14). Imaging examination plays an important role in the diagnosis of retroperitoneal cystic lymphangioma. Ultrasound has a unique advantage in showing the size, location, content and boundary of the cyst. However, when there is hemorrhage and necrosis in the capsule, the echo of the contents will produce some changes, which will affect the judgment. Abdominal enhanced CT is the first choice.Abdominal enhanced CT can be used to observe the density of the tumor to determine the relationship with the surrounding tissue, blood vessels and organs, which can distinguish retroperitoneal lymphangioma from intraperitoneal lymphangioma. Magnetic resonance(MRI) is more sensitive in showing intra-cyst bleeding and content (15–17). However, a final diagnosis still requires pathological biopsy, which is characterized by abnormally dilated lymphatic vessels lined with flat endothelial cells with smooth muscle, blood vessels, fat and lymphatic matrix (18). CT was considered as cystic lymphangioma, and the final diagnosis was confirmed by postoperative pathology.

Without intervention, the volume of cystic lymphangioma will gradually increase, thus oppressing adjacent tissues, blood vessels, nerves, organs and so on, resulting in related complications (19). In our case, the volume increased by about 13 times over a year, leading to the occurrence of this symptom. Surgical resection of tumor is the first choice for the treatment of retroperitoneal lymphangioma. during the operation, attention should be paid to the relationship between tumor and surrounding tissue, blood vessels, nerves and organs, so as to remove the tumor completely and reduce the postoperative recurrence rate (20, 21). Recently some methods of nonsurgical treatment have emerged. Among them, sclerotherapy is the most commonly used alternative therapy for lymphangiomas that cannot be completely resected or diagnosed as difficult to operate. When compared with surgical resection, sclerotherapy has the advantages of being simple and causing less injury. Besides, several studies have reported that sclerosing agents can effectively treat large cystic lymphangioma, even though their curative effect on microcystic lymphangioma is much lower (22–24). Examples of such common hardeners include OK-432, doxycycline, bleomycin, and ethanol. Notably, OK-432 sclerotherapy can not only induce and activate leukocytes to produce cytokines, which in turn increase endothelial cell permeability, accelerate the speed and flow of lymphatic drainage, and facilitate lymphangioma cystic cavity shrinkage and lesion regression, but also reduce complications and focal fibrosis, making it a promising alternative to surgery, especially for patients with microcystic lymphangioma (25, 26). However, sclerotherapy has been reported to cause some adverse reactions, such as airway obstruction and skin necrosis. Therefore, the effectiveness of sclerotherapy needs to be further evaluated (27, 28). Another notable method is radiofrequency ablation, which destroys diseased tissues at low temperatures while causing less damage to the surrounding tissues. Presently, radiofrequency ablation is the first choice of treatment for treating oral and pharynx microcystic lymphangioma (29, 30). Some studies have also shown that radiofrequency ablation is an effective method for the treatment of superficial microcystic lymphangioma; however, further development and research are needed to better apply it for the treatment of microcystic lymphangioma found in other body parts.

Although the above-mentioned methods have greatly helped in the treatment of lymphangioma, clinicians still find it difficult to obtain optimal results for lymphangiomas with large lesion areas and invasive growth. Importantly, recent studies have shown the efficacy of several drugs in the treatment of lymphangioma, and some of these drugs have been used in treating patients with lymphangiomas, such as sildenafil, propranolol,sirolimus, and several inhibitors targeting the PI3K/AKT/mTOR signaling pathway (PI3K inhibitors, AKT inhibitors, MAPK inhibitors, sorafenib, etc.) (31, 32). However, some studies have demonstrated that sildenafil is not effective in the treatment of microcystic lymphangioma, whereas sirolimus has been demonstrated to have good efficacy in the treatment of microcystic lymphangioma and may become the focus of future research. In addition, studies have also shown that BMP and Wnt modulators, calcium channel blockers, and KATP activators (minoxidil) may also have some therapeutic potential (33).

Since a single treatment cannot achieve satisfactory results, clinicians are encouraged to use multiple methods of combined therapy. Consequently, surgical resection, as the main treatment, can be used to remove a significant volume of large cystic lesions, while the remaining diseased tissues can be treated with sclerotherapy. For instance, bleomycin is currently used in the treatment of residual small cystic diseases and has achieved some efficacy (34). However, sometimes lymphangiomas may require several rounds of sclerotherapy. For tumors that are large or widely enclosed and have invaded their surrounding tissues, blood vessels, and nerves, drugs or sclerotherapy can be taken first to reduce the size of the lesions before resecting them through surgery. Alternatively, postoperative sclerotherapy, drugs, radiofrequency ablation, and other combined therapy can be used. Unfortunately, there are currently no unified guidelines for the diagnosis and treatment of lymphangiomas, and further verification is still needed.

We provided a case report and on the basis of literature review, retroperitoneal cystic lymphangioma was investigated.PubMed was searched using these key words: (“Lymphangioma, Cystic” or “Cystic Lymphangioma” or “Cystic Lymphangiomas” or “Lymphangiomas, Cystic” or “Hygroma” or “Hygromas, Cystic” or “Cystic Hygroma Colli” or “Colli, Cystic Hygroma” or “Hygroma Colli, Cystic” or “Hygroma” or “Hygromas”) and (“Retroperitoneal Space” or “Retroperitoneal Neoplasms” or “Retroperitoneal Fibrosis”). Key words referred to medical subject heading (MeSH). And the search terms used on PubMed were: (([“Lymphangioma, Cystic"(Mesh)] OR ((((((((((([Cystic Lymphangioma(Title/Abstract)] OR [Cystic Lymphangiomas(Title/Abstract)]) OR [Lymphangiomas, Cystic(Title/Abstract)]) OR [Hygroma, Cystic(Title/Abstract)]) OR [Cystic Hygroma(Title/Abstract)]) OR [Cystic Hygromas(Title/Abstract)]) OR [Hygromas, Cystic(Title/Abstract)]) OR [Cystic Hygroma Colli(Title/Abstract)]) OR [Colli, Cystic Hygroma(Title/Abstract)]) OR [Hygroma Colli, Cystic(Title/Abstract)]) OR [Hygroma(Title/Abstract)]) OR [Hygromas(Title/Abstract)]))) AND ([Retroperitoneal(Title/Abstract)] OR ((([“ Retroperitoneal Space"(Mesh)] OR “Retroperitoneal Neoplasms"[Mesh]) OR “Retroperitoneal Fibrosis"[Mesh]))).

We systematically reviewed the studies returned from the literature searches.The inclusion criteria were as follows: (I) retroperitoneal cystic lymphangioma; (II) over 18 years old; (III) full text; (IV) manuscripts written in English. Studies were excluded for any of the following: (I) the study was a review, meeting abstract, non-clinical study, or *in vitro* study; (II) inferior quality literature or with insufficient outcome indicators; (III) The lesion is not retroperitoneal. Each abstract is carefully reviewed by two different authors (SS and DS). We conducted a systematic review of 240 records and finally included 29 articles involving 34 cases. Detailed characteristics of the reported cases are provided in supplemental (Table 1) (35–63). We made the statistics on the above date.There were 21 males and 13 females, aged from 25 to 76 years old, with an average age of 45.82 years old. 25 cases underwent laparotomy and 9 cases underwent laparoscopic surgery. The average diameter of tumor was 13.44 cm. Among the 34 patients, 1 case had postoperative complications, 20 cases had no postoperative complications, and the other 14 cases had no related data.

**Table 1:** Characteristics of the adult cases of retroperitoneal cystic lymphangioma reported in the literature.

Auther	References No.	No. Patients	Open/Laparoscopy/Conservative	Male/Female	Age years (Means)	Tumer Size (cm) (Means)	Follow-up (mo)	Recurrence
Shayesteh et al.	(35)	1	1/0/0	0/1	29	29	NP	NP
Dunev et al.	(36)	1	1/0/0	0/1	35	18.5	NP	NP
Nuzzo et al.	(37)	2	2/0/0	2/0	61.5	17.5	15	0
Saadi et al.	(38)	5	5/0/0	1/4	45	14	32.6	0
Lim et al.	(39)	1	1/0/0	1/0	74	9	NP	NP
Kodera et al.	(40)	1	1/0/0	0/1	39	5.5	24	0
Cherk et al.	(41)	1	1/0/0	0/1	41	9	30	0
Bhavsar et al.	(42)	1	1/0/0	1/0	54	12.2	NP	NP
Aminian et al.	(43)	1	1/0/0	1/0	50	NP	NP	NP
Lai et al.	(44)	1	0/1/0	1/0	47	5	NP	0
Liedtke et al.	(45)	1	1/0/0	1/0	61	19	3	0
Kalish et al.	(46)	1	1/0/0	1/0	22	14	NP	NP
Suhani et al.	(47)	1	1/0/0	0/1	22	22	NP	NP
Tripathi et al.	(48)	1	1/0/0	1/0	55	15	12	0
Sato et al.	(49)	1	0/1/0	0/1	30	5	24	0
Chaker et al.	(50)	1	1/0/0	1/0	68	4	50	0
Olaoye et al.	(51)	1	1/0/0	1/0	20	NP	36	0
Rezaee et al.	(52)	1	0/1/0	1/0	27	12.6	1	0
Black et al.	(53)	1	0/1/0	1/0	66	5	1	0
Hubli et al.	(54)	1	1/0/0	1/0	36	40	9	0
Rajput et al.	(55)	1	1/0/0	1/0	65	10	1	0
Kasza et al.	(56)	1	0/1/0	1/0	52	9.6	9	0
Izumi et al.	(57)	1	0/1/0	0/1	76	18	NP	1
Ionescu et al.	(58)	1	1/0/0	1/0	31	14.4	NP	NP
Tsukamoto et al.	(59)	1	0/1/0	0/1	36	10	12	0
Mabrouk et al.	(60)	1	1/0/0	1/0	70	19.5	6	0
Suryawan et al.	(61)	1	0/1/0	1/0	22	9.4	6	0
Chung et al.	(62)	1	1/0/0	1/0	56	4.5	0.3	0
Colovic et al.	(63)	1	0/1/0	0/1	26	5	6	0

Surgery (open, laparoscopy); No. patients, number of patients; NP, not precised.

Compared with the case reports in the existing literature, our case was first found during the physical examination a year ago, and the patient himself did not have obvious symptoms of discomfort, so there was no follow-up treatment. Preoperative abdominal contrast-enhanced CT showed that the case was a huge retroperitoneal cystic mass of approximately 24.2 cm × 16.7 cm × 27.8 cm. Up to the lower edge of the pancreas, down to the peritoneal reflex, left and right to the two abdominal walls, anterior to the anterior abdominal wall, and back to the front of the spinal lamina, occupying most of the abdominal cavity and pelvic cavity, squeezing the small intestine and oppressing the inferior vena cava. The nature can not be clear, consider the possibility of benign, but do not rule out the possibility of malignant. This time, due to the increase in the size of the cyst, it oppresses the surrounding intestines, causing the diaphragm to move upward, and some cysts rupture and bleeding, which irritates the intestinal tract, resulting in acute abdominal pain, abdominal distension, intestinal obstruction and dyspnea. Because of the critical condition, we decided to carry out exploratory laparotomy. During the operation, a huge retroperitoneal cystic mass was seen, which almost occupied the whole abdominal cavity and pelvic cavity. We found that the tumor was closely wrapped and adhered to the abdominal aorta, superior mesenteric artery, inferior mesenteric artery, inferior vena cava and other blood vessels, and pressed the surrounding intestinal lumen. Part of the tumor ruptured and bleeding could be seen on the surface. As the nature of the tumor was not clear, we decided to remove the tumor as completely as possible. The surgeon uses scissors to separate carefully and sharply to minimize the rupture and bleeding of the tumor and blood vessels. Accurate and meticulous operation during sharp separation of enclosing and adherent blood vessels to reduce the risk of hemorrhagic shock. When the tumor is completely removed, it is removed slowly to avoid sharp fluctuations in blood pressure. The patient recovered well after operation and was discharged smoothly 10 days later.

However, there are still some areas that need to be improved in our program. for example, in this operation, we can first extract the contents of the cyst to reduce the volume of the tumor, reduce the difficulty of the operation, and shorten the duration of the operation. it may make the patient recover faster and better. Secondly, we only use a single surgical resection, sometimes can not achieve satisfactory results, we should use a variety of methods combined treatment, surgical resection as the main treatment, and the remaining lesions can be treated with sclerotherapy. in addition, for invasive and recurrent people, targeted therapy is also essential.

The purpose of this case is to share our process of diagnosis and treatment of this rare abdominal cystic mass, and to provide some help for relevant people, institutions and fields, but the combined treatment of cystic lymphangioma is still lack of unified understanding. we still need to further explore.

## Conclusion

In this case, we report a patient with giant retroperitoneal cystic lymphangioma who underwent exploratory laparotomy combined with preoperative abdominal CT and acute abdominal pain, abdominal distension and dyspnea. Because of the large volume of the tumor and its close relationship with the superior mesenteric artery and other blood vessels, the surgeon used scissors to separate the tumor sharply and removed the whole tumor completely.

## Data Availability

The original contributions presented in the study are included in the article/Supplementary Material, further inquiries can be directed to the corresponding author/s.
